# Fibrous configuration of the fascia iliaca compartment: An epoxy sheet plastination and confocal microscopy study

**DOI:** 10.1038/s41598-020-58519-0

**Published:** 2020-01-31

**Authors:** Zhaoyang Xu, Bin Mei, Ming Liu, Lili Tu, Han Zhang, Ming Zhang

**Affiliations:** 1 Department of Anatomy, Anhui Medical University, Hefei, China; 2 Department of Anatomy, University of Otago, Dunedin, New Zealand; 3 Department of Anaesthesiology, First Affiliated Hospital of Anhui Medical University, Hefei, China; 4 Department of Ultrasound, Taian Chinese Traditional Medicine Hospital, Taian, China; 5 School of Medicine, University of Otago, Dunedin, New Zealand

**Keywords:** Musculoskeletal system, Nervous system

## Abstract

**Background and Objectives:** The underlying anatomical mechanism of the ultrasound-guided fascia iliaca compartment (FIC) block for anaesthesia and analgesia in the lower limb has not been illuminated and numerous variations were attempted to achieve an optimal needle placement. This study aimed to define the fibrous configuration of the FIC. **Methods:** A total of 46 adult cadavers were studied using dissection, latex injection, epoxy sheet plastination and confocal microscopy. **Results:** (1) The fascia iliaca originated from the peripheral fascicular aponeurotic sheet of the iliopsoas. (2) The FIC was a funnel-shaped adipose space between the fascia iliaca and the epimysium of the iliopsoas, had a superior and an inferior opening and contained the femoral and lateral femoral cutaneous nerves but not obturator nerve. (3) The estimated volume of the FIC in the cadavers was about 23 mls, of which about one third was below the level of the anterior superior iliac spine. **Conclusions:** This study revealed that the fascia iliaca was aponeurotic and may be less permeable for the local anesthetics. **Conclusions:** The FIC contained only the femoral and lateral femoral cutaneous nerves and communicated with the extraperitoneal space and femoral triangle adipose space via its superior and inferior opening, respectively.

## Introduction

The fascia iliaca compartment block (FICB) has been used for anaesthesia and analgesia in knee and hip surgery^1^ and acute pain management^2^. The FICB technique is established on a hypothesis that the femoral, lateral femoral cutaneous (LFCN), genitofemoral and obturator nerves lie close together within the same fascial envelope, namely the fascia iliaca compartment (FIC)^1,3^, thus its key technical point is to deliver the local anaesthetic (LA) into this fascial envelope. However, the suggested practical procedures vary widely among published descriptions of the FICB technique^4^.

The main controversies focus on the optimal needle placement site^2,5,6^ and distribution pattern and volume of the LA^7–9^. These issues are closely related to precise understanding of the fascial configuration of the FIC. For example, the classical needle placement was below the inguinal ligament^1^ whereas several recent studies reported that a supra-inguinal injection had an advantage of more dorsal and proximal spread of the LA in the FIC with a higher block success^2,6,10^. Using magnetic resonance imaging (MRI), Swenson *et al*.^11^ compared the distribution pattern of the LA spread between the ultrasound-guided FICB and “3-in-1” block, and found a consistent superior spead of the LA to the level of the retroperitoneal adipose space in both techniques. However, earlier studies with evaluation of radiography or MRI did not observe any superior spread of the LA during the “3-in-1” block^1^. Anatomically, the origin of the fascia iliaca was defined as either the aponeurotic sheet^12^ or condensation of the extraperitoneal tissue^13^. Thus, the underlying anatomical mechanism of the FICB remains to be illuminated, and there are few solid anatomical data to verify the original hypothesis of the FICB technique: the femoral, LFCN, genitofemoral and obturator nerves lie close together within the same fascial envelope^3^.

Technically, fascia-like structures are so pervasive and interconnected and are easily damaged or altered during preparation or during surgery^14^. The newly-developed epoxy sheet plastination technology in combination with confocal microscopy not only preserves the *in situ* position of bones, cartilages and soft tissues without decalcification but also allows these structures to be examined undisturbed in their natural state both at macroscopic and microscopic levels^15–17^. In order to clarify the controversies related to the optimal needle placement site and the distribution and volume of the LA during the FICB, this study aimed to to define the three-dimensional (3D) fibrous configuration of the FIC.

## Results

### Fascial configuration below the inguinal ligament

#### Origins of the fascia lata

**Figure 1:**
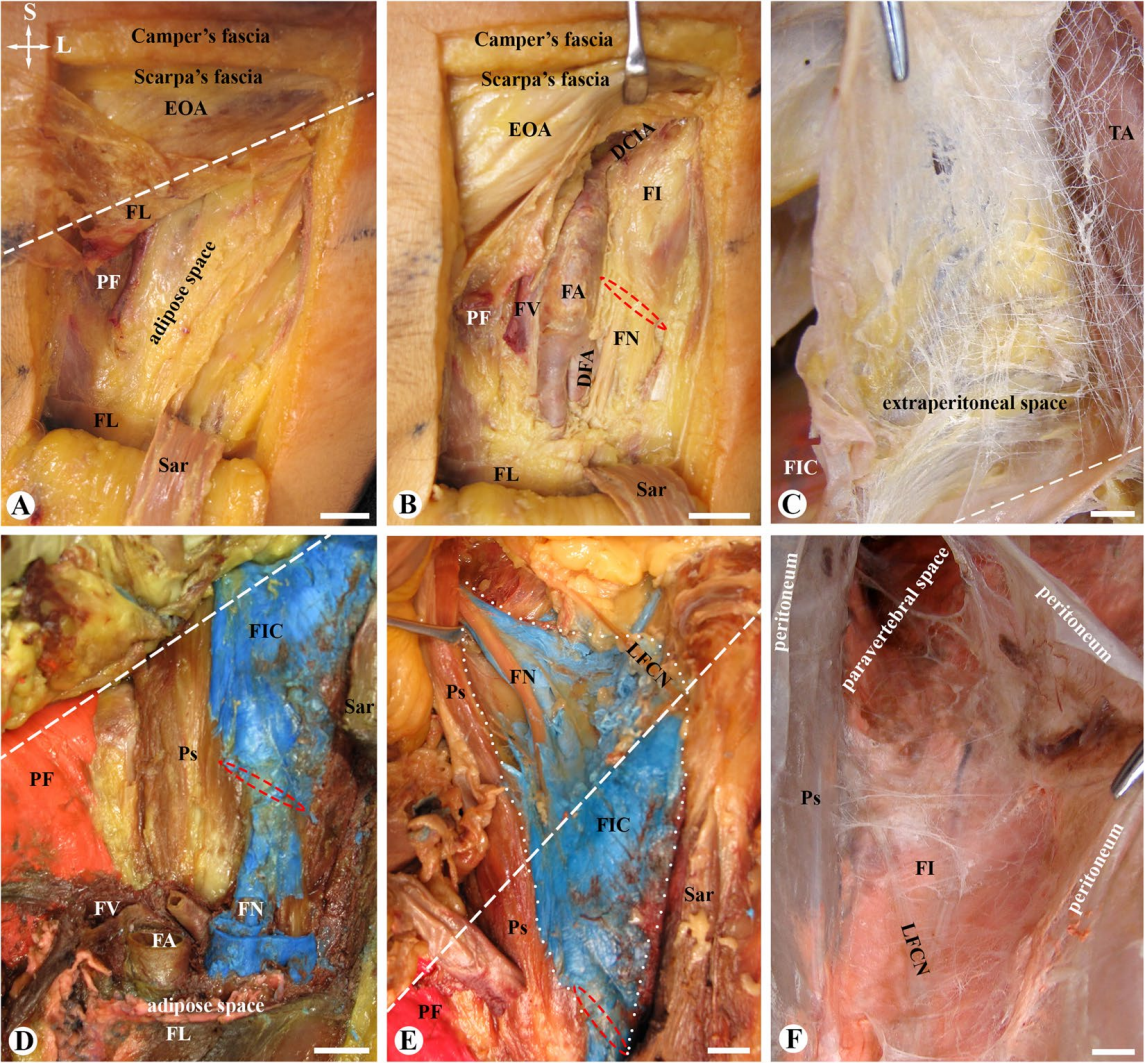
A layer-by-layer dissection (**A**,**B**) and latex injection (**C–F**) in the femoral triangle. (**A**) The adipose space underneath the fascia lata (FL) and sartorius (Sar; reflected inferiorly). EOA: external oblique abdominis; PF: pectineal fascia. (**B**) The femoral nerve (FN) pierces the fascia iliaca (FI) via the inferior opening (red dashed circle) of the fascia iliaca compartment (FIC). DCIA: deep circumflex iliac artery; DFA: deep femoral artery; FA: femoral artery; FV: femoral vein. (**C**) Single-coloured latex injected underneath the fascia iliaca, showing latex spread in the FIC but not in the extraperitoneal space between the transverse abdominis (TA) and peritoneum (held by forceps). D and E: Coloured Latex injected underneath the fascia lata (pink), fascia iliaca (blue) and pectineal fascia (red) prior to fascia dissection. Pink latex spreads in the adipose space superficial and lateral to the femoral artery (FA) and vein (FV). Blue latex (about 20 mls) fills in the FIC (white dotted curves) and spreads through its inferior opening (red dashed circle). F: Single-coloured latex (more than 30 mls) injected underneath the fascia iliaca (FI), showing latex spread in the FIC and paravertebral space. Dashed lines: level of the inguinal ligament; Ps: psoas; Ili: iliacus; LFCN: lateral femoral cutaneous nerve; Crossed-arrows: orientation of the figure (L: lateral; S: superior); Bars = 10 mm.

**Figure 2:**
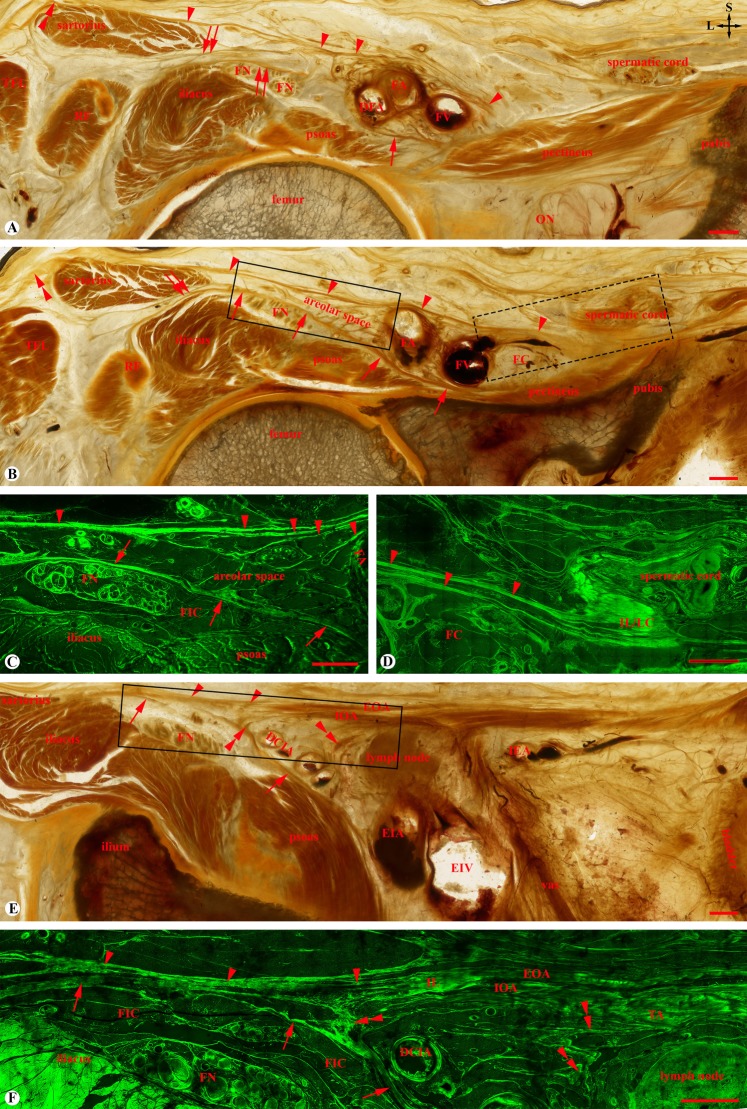
Fascia iliaca compartment on three adjacent transverse sections at the levels of the medial (**A**), middle (**B**) and lateral (**E**) thirds of the inguinal ligament, respectively. (**A**) The fascia iliaca appears as a medial psoas part (single arrow) which contributes to the posterior wall of the femoral vascular sheath, and a lateral conjoint tendinous sheet part (double arrows). Single arrowheads: fascia lata originated from the inferior prolongation of the inguinal ligament; Double arrowheads: fascia lata originated from the iliolata ligaments; DFA: deep femoral artery; FA: femoral artery; FN: femoral nerve; FV: femoral vein; ON: anterior division of the obturator nerve; RF: rectus femoris; TEF: tensor fascia lata. (**B–D**) The intact fascia iliaca (single arrows) covers the femoral nerve (FN), laterally receives aponeurotic fibers (double arrows) from the iliacus, rectus femoris (RF) and sartorius, and medially merges with the pectineal fascia. FC: femoral canal. (**C**,**D**) are the mirror confocal images of the solid and dashed line boxes in (**B**) showing the multiple-layered fascia lata (single arrowheads) originated from the inferior prolongation of the inguinal (IL) and lacunar ligaments (LC) and contributed to the anteromedial wall of the femoral vascular sheath. The epineurium of the femoral nerve (FN) is within the fascia iliaca compartment (FIC). (**E**,**F**) Aponeurotic fibers of the psoas and iliacus (single arrows), and the internal oblique abdominis (IOA) and transverse abdominis (TA) (double arrowheads) are intermingled to form the conjoint tendinous sheet of the fascia iliaca. (**F)** is the mirror confocal image of the box in (**E**) showing the continuation of the inguinal ligament (IL), its inferior prolongation (single arrowheads) and external oblique (EOA), internal oblique (IOA) and transversus (TA) abdomines. EIA/EIV: external iliac artery and vein; DCIA: deep circumflex iliac artery; IEA: inferior epigastric artery; vas: ductus deference. Crossed-arrows: orientation of the figure (L: lateral; S: superior); Bars = 5 mm.

**Figure 3:**
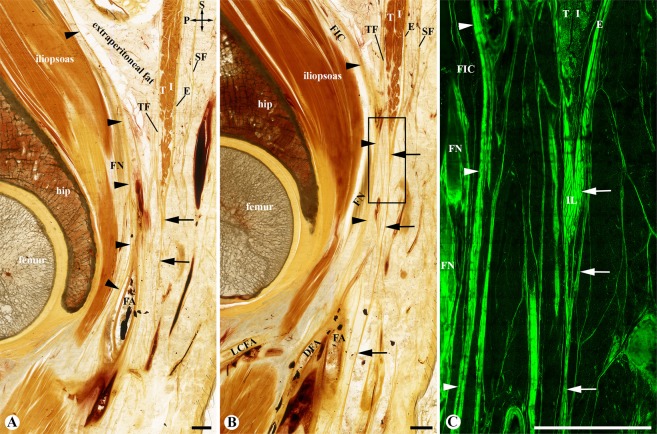
The origin of the fascia lata and its relationship with the fascia iliaca and femoral nerve. (**A**,**B**) two adjacent sagittal sections at the level of the hip joint; (**A)** is medial to (**B**,**C**) the mirror confocal image of the box in (**B**) The aponeurotic fibers from the external oblique (E), internal oblique (I) and transversus (T) abdomines form the inguinal ligament (IL) and its inferior prolongation which contributes the fascia lata (arrows). The fascia iliaca (arrowheads) borders the fascia iliaca compartment (FIC), covers the femoral nerve (FN) and contributes to the posterior wall of the femoral vascular wall. DFA: deep femoral artery; FA: femoral artery; LCFA: lateral circumflex femoral artery; SF: Scarpa’s fascia; TF: transversalis fascia; Crossed-arrows: orientation of the figure (P: posterior; S: superior); Bars = 5 mm.

The fascia lata in the femoral triangle had two origins. The medial part of the fascia lata was the inferior prolongation of the inguinal ligament, which contributed to the anterior and medial walls of the femoral vascular sheath (Figs. 1A, 2A–1F and 3A–C) and fused with the pectineal fascia (Figs. 1A, 2B,D). The inferior prolongation of the inguinal ligament originated from the aponeurotic fibres of not only the external oblique abdominis but also the internal oblique and transversus abdominis, and thus appeared as a 2 or 3 layered fascia anteriorly to the femoral nerve (Figs. 2C,D, 3B,C). The lateral part of the fascia lata overlapped the sartorius and was formed by 2–3 curtain strip-like ligaments which were termed “the iliolata ligaments” in this study (Fig. 2A,B)^18^. The iliolata ligaments superiorly inserted to the ASIS, inferiorly and medially contributed to the fascia lata, and laterally and superficially fanned out and continued as skin ligaments in the subcutaneous tissue.

#### Fascia-like structures underneath the fascia lata

In addition to areolar tissue, there were two groups of fascia-like structures deep to the fascia lata: a lateral group with multiple transversely or obliquely orientated fascial layers which originated from the aponeurotic fibres of the sartorius and rectus femoris (Fig. 2A,B) and a medial group with longitudinally orientated fibrous bundles (Fig. 2B,C) which originated mainly from the aponeurotic fibres of the transversus abdominis (Fig. 2E,F), internal oblique abdominis and external oblique abdominis (Fig. 2C).

### Origin and configuration of the fascia iliaca

**Figure 4:**
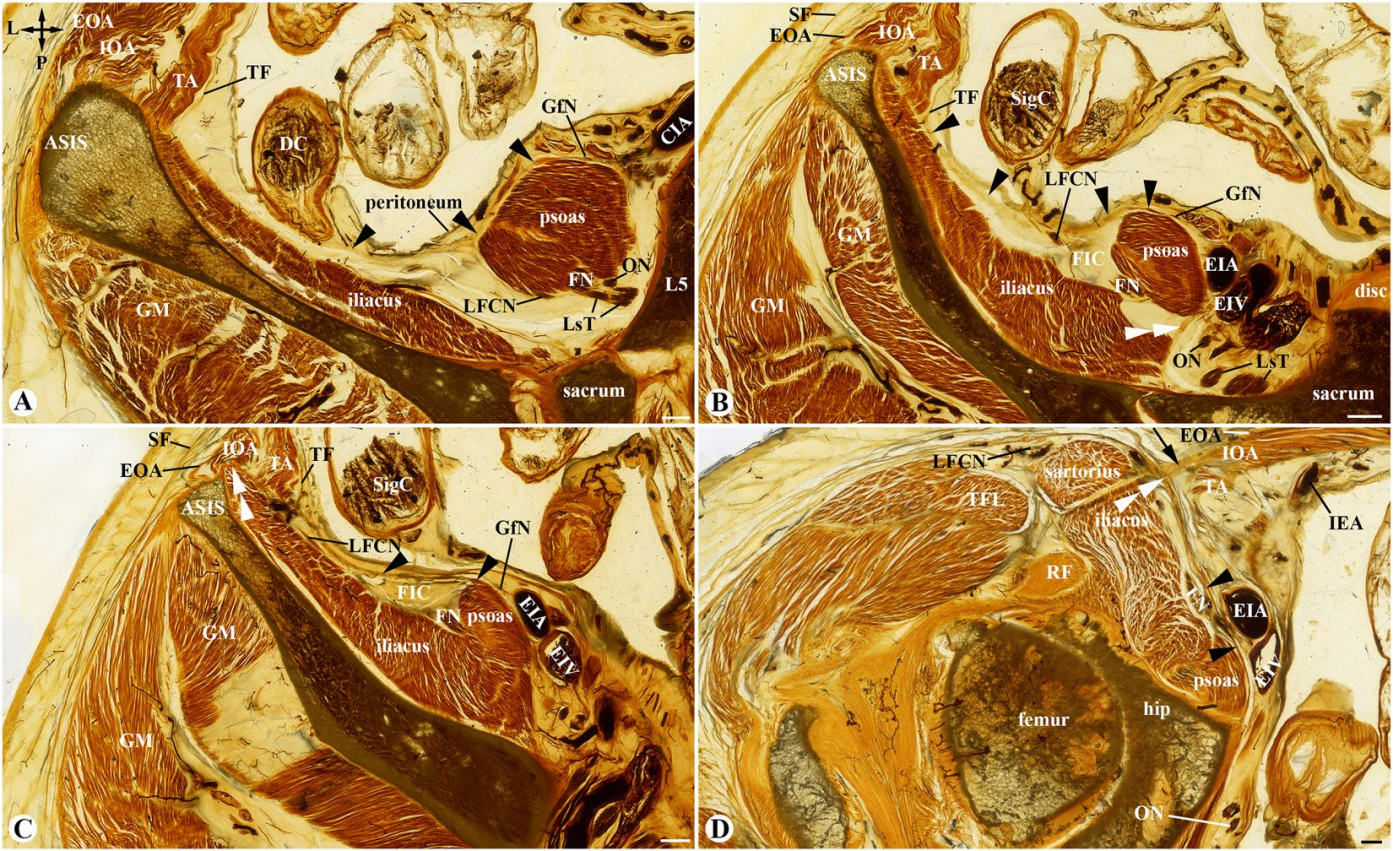
The fascia iliaca compartment on four adjacent transverse sections above the inguinal ligament. (**A**) A section at the 5^th^ lumbar vertebral level (L5) showing that the peripheral fascicular aponeurotic sheet (single arrowheads) of the psoas anchors at the descending mesocolon (DC) borders the paravertebral areolar gutter which contains the femoral nerve (FN), lateral femoral cutaneous nerve (LFCN), obturator nerve (ON) and lumbosacral trunk (LsT) and communicates anteriorly with the extraperitoneal areolar space which is medial to the epimysium of the iliacus and posterior to the transversalis fascia (TF). ASIS: anterior superior iliac spine; CIA: common iliac artery; GfN: genitofemoral nerve; EOA: external oblique abdominis; IOA: internal oblique abdominis; TA: transversus abdominis; GM: gluteal muscle. (**B**) An adjacent section 3.4 cm inferior to (**A**) showing that the peripheral fascicular aponeurotic sheet (single arrowheads) of the psoas forms the fascia iliaca and borders the fascia iliaca compartment (FIC) which contains the FN and LFCN, but not the ON and GfN. Double arrowheads point to a membrane-like structure separating the FIC from the paravertebral gutter. EIA/EIV: external iliac artery and vein; disc: 5^th^ lumbar intervertebral disc; SF: Scarpa’s fascia; SigC: sigmoid colon. (**C**) An adjacent section 2 cm inferior to (**B**) showing that the internal oblique abdominis (IOA), transversus abdominis (TA) and transversalis fascia (TF) fuse with aponeurotic fibers of the iliacus, forming a conjoint tendinous sheet (double arrowheads) of the fascia iliaca (single arrowheads). (**D**) An adjacent section 4 cm inferior to (**C**) showing that the conjoint tendinous sheet (double arrowheads) of the fascia iliaca (single arrowheads) below the ASIS is strengthened by the inferior prolongation of the inguinal ligament (single arrow), and the fascia lata over the sartorius. Crossed-arrows: orientation of the figure (L: lateral; P: posterior). Bars = 5 mm.

The fascia iliaca was formed by the peripheral fascicular aponeurotic sheets (PFAS) of the psoas (Fig. 4B,C) and iliacus (Fig. 2A,B), rather than the epimysium of the muscles. Its anteroinferior part was further enhanced by the aponeurotic fibres of the transversus abdominis and internal oblique abdominis (Fig. 2E,F).

Superiorly, the fascia iliaca gradually disappeared at the level of about the 5^th^ lumbar vertebra (L5) where was the uppermost origin of the iliacus muscle. Above the L5 level, the PFAS of the psoas anchored at the attachment site of the mesocolon (Fig. 4A), and below the L5 level, was continuous anteromedially with the transversalis fascia (Fig. 4B). Posteriorly, the PFAS of the psoas extended to the posterior edge of the iliacus, forming a membrane-like structure (Fig. 4B). This membrane-like structure was a small triangular aponeurotic septum to separate the FIC from the paravertebral space (Fig. 4B) and disappeared after the two muscles united (Fig. 4C). It also separated the obturator nerve from the FIC. Thus, the superior opening of the FIC was at the L5 level and the FIC contained only the femoral nerve and LFCN (Fig. 4B) but not the obturator nerve. Via the superior opening, the FIC communicated with the extraperitoneal and paravertebral adipose space (Fig. 4A).

At the level of the lateral third of the inguinal ligament, the fascia iliaca was strengthened by the aponeurotic fibres of the transversus abdominis and internal oblique abdominis (Fig. 2E,F), forming a conjoint tendinous sheet (Fig. 4C,D). At the level of the middle third of the inguinal ligament (Figs. 2B,C and 3A–C), the fascia iliaca fused medially with the pectineal fascia and was strengthened laterally by the PFAS of the iliacus and rectus femoris. At the level of the medial third of the inguinal ligament (Fig. 2A), the femoral nerve pierced through the conjoint tendinous sheet, creating an inferior opening of the FIC. Laterally to the nerve, the conjoint tendinous sheet loosened and appeared as multiple spur-like structures (Fig. 2A).

### Configuration of the fascia iliaca compartment

The FIC was an adipose space between the fascia iliaca and the epimysium of the iliopsoas (Figs. 2 and 4). The FIC communicated anteromedially with the extraperitoneal space and posteriorly with the paravertebral space via its superior opening at about L5 level (Fig. 4A). The FIC and the psoas compartment which was between the psoas and quadratus lumborum were not enclosed within a same fascial envelope. Inferiorly, the FIC gradually tapered and communicated with the adipose space underneath the fascia lata via its inferior opening, the exit of the femoral nerve in the conjoint tendinous sheet (Fig. 2A).

The FIC contained the femoral nerve and LFCN, but not the oberturator and genitofemoral nerves (Fig. 4B). The femoral nerve traversed the FIC and had an intact epineurium (Fig. 2C,F), whereas the LFCN pierced the conjoint tendinous sheet at the level of the ASIS (Fig. 4C,D).

**Figure 5:**
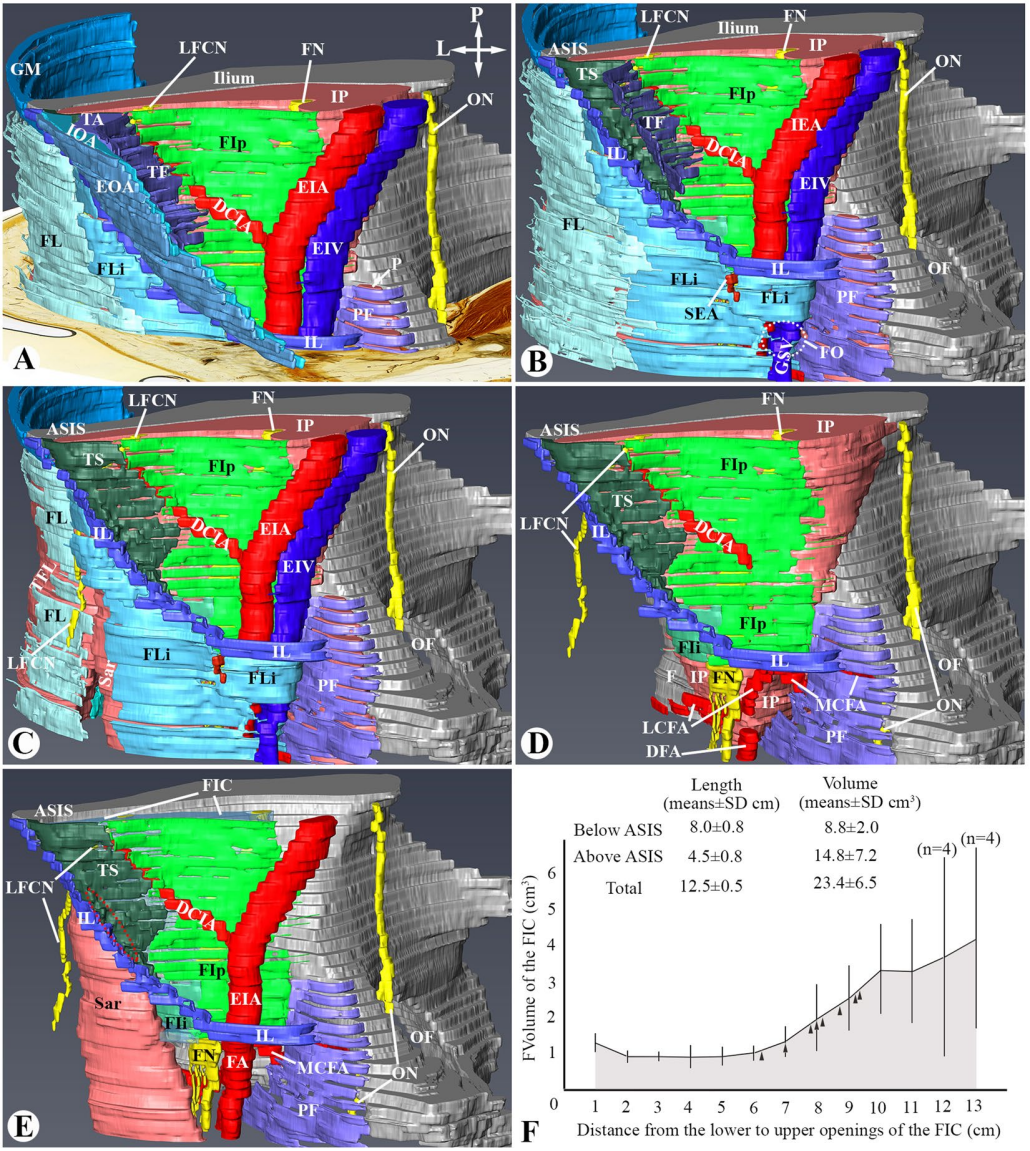
A three-dimensional (3D) image reconstructed from the plastinated sections showing the architecture of the fascia iliaca below the anterior superior iliac spine (ASIS). (**A**) The 3D image with a plastinated section. Crossed-arrows: orientation of the figure (L: lateral; P: posterior); DCIA: deep circumflex iliac artery; EIA/EIV: external iliac artery and vein; EOA: external oblique abdominis; FIp: psoas part of the fascia iliaca; FL: fascia lata; FLi: fascia lata formed by the inferior prolongation of the IL; FN: femoral nerve; GM: gluteal muscles; IL: inguinal ligament; IOA: internal oblique abdominis; IP: iliopsoas muscle; LFCN: lateral femoral cutaneous nerve; ON: obturator nerve; P: pectineus muscle; PF: pectineal fascia; TA: transversus abdominis; TF: transversalis fascia. (**B**) The conjoint tendinous sheet (TS) is the anterior part of the fascia iliaca, inferior to the ASIS, anterior to the transversalis fascia (TF), and posterior to the IL. Dashed circle and FO: fossa ovalis; GSV: great saphenous vein; SEA: superficial epigastric artery. See Supplemental Digital Content 1 for a 3D view of (**B)**. (**C**) The lateral part of the FL is partially removed to expose the LFCN which pierces the TS and runs under the IL. Sar: sartorius; TEL: tensor fascia lata. (**D**) The fascia iliaca consists of three parts - FIp, TS and FIi, and pierced by the FN and LFCN below the IL. DFA: deep femoral artery; F: femur; LCFA: lateral circumflex femoral artery. (**E**) An optimal needle placement (dashed circle) for the ultrasound-guided FIC block is through the TS at the site inferior to the ASIS and medial to the upper attachment of the sartorius (Sar). See Supplemental Digital Content 2 for a 3D view of (**E)** without bones. (**F**) The average length and volume of the FIC estimated in the plastinated slices from 8 sides of 4 cadavers. The arrowheads in the graph indicate the level of the ASIS in the individual sides.

The average volume of the FIC was 23.4 ± 6.5 cm^3^, of which about one-third (8.8 ± 2.0 cm^3^) was below the ASIS. The distance from the ASIS to the exit of the femoral nerve was 8.0 ± 0.8 cm (Fig. 5F).

To test the spread pattern within the various fascial compartments in the region, fascia-like structures were exposed by a layer by a layer dissection (Fig. 1A,B) and coloured latex was injected underneath a given fascia-like structure (Fig. 1C). The results of the single injection of latex indicated that the fascia lata, fascia iliaca and pectineal fascia singnificantly limited the spread of the injected latex. To reveal the relationship among these three fasciae, the latex with three different colours was injected underneath the fascia lata, fascia iliaca and pectineal fascia, respectively (Fig. 1D,E). No mixture of the injected latex with three different colours was observed.

Underneath the fascia lata, the injected latex spread in multiple layered structures over and lateral to the femoral artery (Fig. 1D). When injected underneath the fascia iliaca with less than 20 mls (Fig. 1E), the latex spread over the iliacus, fully enclosed the femoral nerve and LFCN, and extended superiorly into a narrow gutter between the iliacus and psoas muscles (Fig. 1E) and inferiorly through a narrow canal to the adipose space under the fascia lata (Fig. 1D). When injected underneath the fascia iliaca with 30 mls of latex, the latex spread superiorly into the paravertebral and extraperitoneal spaces above the level of the superior origin of the iliacus muscle. The spread of latex underneath the pectineal fascia was limited to the surface of the pectineus (Fig. 1D).

## Discussion

This study used a well-established novel anatomical technology – a combination of epoxy sheet plastination and confocal microscopy, to reveal the fine fibrous configuration of the fascia iliaca, and its relationship with the FIC and the main branches of the lumbar nerve plexus. To help better understand and interpret those sectional images and conceptualise the optimal needle placement for the FICB, the 3D architecture of one right FIC was reconstructed from 64 serial transverse sections (Fig. 5A–E). Figure 5 illustrates three principal findings of this study. (1) The fascia iliaca originates from the PFAS of the iliopsoas muscle and its anterior part intermingles with aponeurotic fibres of the internal oblique abdominis and transversus abdominis, forming a conjoint tendinous sheet. (2) The FIC is a funnel-shaped adipose space between the fascia iliaca and the epimysium of the iliopsoas muscle, and has a superior and inferior opening via which it communicates with the extraperitoneal space superiorly and an adipose space in the femoral triangle inferiorly. (3) The estimated volume of the FIC in the cadaver was about 23 mls, of which about one third was below the level of the ASIS.

Since its first edition in 1858^12^, Gray’s Anatomy defines that the fascia iliaca is connected laterally to the iliac crest and medially to the pelvic brim, forming the FIC^19^. However, the origin of the fascia iliaca was described as either the aponeurotic layer^12^ or condensation of the extraperitoneal adipose tissue^13^. The present study supported the original description of Gray’s Anatomy^12^ and precisely demonstrated that its origin was the PFAS of the psoas and iliacus muscles predominantly above and below the inguinal ligament, respectively. As showed in the latex spread tests of this study (Fig. 1C–F), the aponeurotic fascia appears less porous than a fascia formed by condensation of the loose connective tissue.

Superiorly, the fascia iliaca disappeared at the L5 level where the uppermost part of the iliacus muscle arises from the ilium, and the PFAS of the psoas anchors at the mesocolon attachment rather than continues with the transversalis fascia. Thus, the fibrous configuration of the fascia iliaca is different from the psoas fascia and the level of the uppermost iliacus muscle should be defined as the superior opening of the FIC. Via the superior opening, the FIC communicated freely with the extraperitoneal space and the paravertebral space. Below the superior opening, the fascia iliaca formed an aponeurotic fascial barrier to separate the FIC from extraperitoneal space and paravertebral space. The FIC was not same as the psoas compartment which was between the psoas and quadratus lumborum^20,21^.

It has been proposed that inconsistent results of anaesthetic efficacy of the FICB may result from relatively minor variations in needle placements^5,7,8,11^. For example, the classic FICB inserts the needle at the level of the lateral third of the inguinal ligament and does not directly target the femoral nerve^1^, whereas the “3-in-1” block places the needle 1 cm below the middle third of the inguinal ligament and 1 cm lateral to the femoral artery, and targets the femoral nerve^3^. Although both the FICB and “3-in-1” block appear to be equally effective to block the femoral nerve, the results of the present study suggest that the underlying anatomical basis of their equal effectiveness may result from a firm compression distal to the site of injection, which is suggested by both techniques. The firm compression distal to the site of injection may confine the LA within the FIC and guide its superior spread, and in the “3-in-1” block, may drive some LA into the FIC via its inferior opening.

The distribution pattern of the LA is closely related to the injected volume. As estimated in the present study, the total volume of the FIC in the adult cadaver was about 23 ml, of which one third (about 8 ml) was below the ASIS. According to the literature, the volume of the LA commonly used in the FICB or “3-in-1” block ranges from 15 to 40 ml^8,22–24^. Therefore, if the LA is precisely deposited within the FIC, even with a very small amount (e.g. 7 ml in a case report^25^), the femoral nerve and LFCN can be blocked. More than 20 ml of the LA may overflow the superior opening of the FIC into the paravertebral space to block the obturator nerve and other branches of the lumbar plexus.

One of the difficulties in the ultrasound-guided FICB is poor imaging of the fascia iliaca^6^. The conjoint tendinous sheet of the fascia iliaca described in the present study can be used as a key ultrasound landmark to localize the FIC as it was easily visualized on the surface of the iliacus muscle at the level around and below the ASIS. Several recent reports suggest that a supra-inguinal injection had an advantage of more dorsal and proximal spread of LA in the FIC with a higher block success^2,6,10,24,26^. The ultrasound-guided supra-inguinal FICB technique recommends that the puncture point is medial to the sartorius which origin is at the ASIS, and the injection point is at the level of the deep circumflex iliac artery which runs paranal to the inguinal ligament. Thus, its injection point is above the inguinal ligament but below the level of the ASIS; the conjoint tendinous sheet can be used as an ultrasound landmark for both supra- and infra-inguinal FICB. In comparison with the classic FICB, the main drawbacks of a supra-inguinal injection may be (1) the mis-injection of the LA into the extraperitoneal space^6,27^ and (2) difficulty to avoid puncturing the peritoneum, because the orientation of the fascia iliaca above the inguinal ligament is almost vertical (Fig. 4A–C) rather than transverse (Fig. 4D).

This study has at least two limitations. Firstly, the number of the cadavers for the estimation of the FIC volume was small and they were from the elderly cadavers and may not be representative for the population, particularly for the living subject. The estimation was based on plastinated slices in which both soft (e.g. fascia, ligaments, tendons, muscles, fat) and hard (e.g. bones, cartilages) tissues are preserved the *in situ* position and the FIC boundaries can be well defined under a microscope. Thus, the results of the quantitative estimation in this study may be different from other classical cadaveric studies, such as the measurement of the volume of injected latex. Secondly, due to the cadaveric model and injected solution characteristics, the few reference spread patterns obtained after injecting under different fascial layers cannot be considered a resemblance of a clinical block.

In summary, this study provides morphological evidence to reveal the underlying anatomical mechanism of the FICB which may help to interpret, evaluate and adjust the femoral nerve block techniques. The latex spread pattern and quantification of the FIC volume tested in this study suggested that with a less than 20 mls of the LA, the FICB may simultaneously block the femoral nerve and LFCN but not the obturator nerve.

## Methods

A total of 46 cadavers (22 females, 24 males; age range, 38−97 years) were studied. These cadavers were embalmed using an ethanol-based mixture and had no know history of pelvis and thigh injury or surgery and no records nor signs (e.g. scars) upon examination indicating pathology. The causes of death included xcardiovascular diseases (17 cadavers), pulmonary diseases (14 cadavers), colorectal or esophageal cancer (11 cadavers) and brain injury (4 cadavers). The cadavers assigned to this project were bequeathed for medical education and research purposes with the written informed consent from the donor or the next of kin as obtained under the Human Tissue Act. The study was performed in accord with our institutional ethical guidelines and approved by the Human Research Ethics Committee in University of Otago for the cadaver study (H18/027).

### Cadaveric dissection

Thirty-eight cadavers were used to expose the femoral nerve and its surrounding fascia-like structures by a layer by layer dissection (Fig. 1A,B) and test the latex spread pattern under a given fascia-like structure in the region with a single injection of coloured latex (8 cadavers; Fig. 1C,F) underneath the fascia-like structure through a 25 G needle. In 3 cadavers, latex with different colours was injected underneath the fascia lata, fascia iliaca and pectineal fascia, respectively, before the fascia was dissected and opened (Fig. 1D,E). The injected volume of latex varied, about 20 to 30 mls in the fascia lata or fascia iliaca and 10 mls in the pectineal fascia. The injection was stopped when there was leakage through the defined fascia. The extent of the injected latex and pressure was not quantified in this study.

### Epoxy sheet plastination and confocal microscopy

A series of transverse (4 sets) and sagittal (4 sets) plastinated slices from 8 cadavers had been pre-prepared over the last two decades for use in various research projects. The detailed procedure of the epoxy sheet plastination technique was recently reviewed by Ottone *et al*.^28^. In brief, the epoxy plastination process entailed inially freezing the specimens at −80 °C for seven days. The specimen was then cut into 2.5 mm-thick serial sections using a diamond band saw (Dramet, Kleinmaischeid, Germany) resulting in a loss of 0.9 mm of slice thickness due to sectioning. The slices were subsequently dehydrated in acetone at −30 °C over a period of four weeks after which they were degreased in acetone at 22 °C−24 °C for three weeks, followed by impregnation with an epoxy resin mixture of E12/E1/AE20/AE30 (Biodur, Heidelburg, Germany) and cured at 45 °C for 5 days. The plastination process results in collagen, elastin, myofilaments and neurofilaments being endogenously auto-fluorescent under the 488 nm excitation. The relevant plastinated sections were examined under a confocal laser scanning microscope (Nikon, Tokyo, Japan). The thickness of the optical section was set at 16.7 µm under a 10x objective.

### Quantification of the fascia iliaca compartment

The volume and length of the FIC was estimated in 8 sides of 4 cadavers which were prepared as the transverse plastinated slices. The length of the FIC was estimated by multiplying the thickness of the section and the interval by the total number of the sections containing the FIC. The total volume of the FIC was estimated by multiplying the average area of the FIC of the sampled sections by the length of the FIC. The area of the FIC on the sampled section was measured using Thermo Scientific Amira software (Amira 6.5.0 version, Thermo Fisher Scientific, Hillsboro, USA. https://www.fei.com/software/amira/).

### Three dimensional architecture of the fascia iliaca compartment

To qualitatively demonstrate 3D architecture of the FIC, 64 images from the right side of a series of plastinated sections were used. The FIC and its surrounding structures were manually segmented as 2D images, reconstructed and displayed as 3D images (Supplemental Digital Contents 1 and 2) in Thermo Scientific Amira software.

### Statistical analysis

Quantitative data were analysed with the SPSS version 25 (IBM corporation, New York, USA) and reported as means ± standard deviation.

## Supporting Information

Supporting Information1.
Supporting Information2.
